# Apoptotic Impact of Heliox Cold Plasma on a Cervical Cell Line Using Gold Nanoparticle-Doped Graphene Oxide Nanosheets

**DOI:** 10.5812/ijpr-150385

**Published:** 2024-11-12

**Authors:** Mahsa Vatani, Simzar Hosseinzadeh, Amirhossein Sari, Hamidreza Ghomi Marzdashti, Azam Rahimpour, Roya Fattahi

**Affiliations:** 1 Plasma Physics Research Center, Science and Research Branch, Islamic Azad University, Tehran, Iran; 2 Medical Nanotechnology and Tissue Engineering Research Center, Shahid Beheshti University of Medical Sciences, Tehran, Iran; 3 School of Advanced Technologies in Medicine, Shahid Beheshti University of Medical Sciences, Tehran, Iran; 4 Laser and Plasma Research Institute, Shahid Beheshti University, Tehran, Iran; 5 Department of Tissue Engineering and Applied Cell Sciences, Faculty of Advanced Technologies in Medicine, Mazandaran University of Medical Sciences, Sari, Iran

**Keywords:** Cervical Carcinoma, Cold Atmospheric Plasma, Cancer, Gold Nanoparticles, Radical Species, Graphene Oxide

## Abstract

**Background:**

Invasive cervical cancer is recognized as the second most common malignancy in women after breast cancer.

**Objectives:**

This study investigates, for the first time, the effect of gold nanoparticle-doped graphene oxide (GO) nanosheets on the human epithelial carcinoma (HeLa) cell line in the presence of heliox cold plasma.

**Methods:**

Graphene oxide nanosheets were synthesized using the Hummer method and then doped with gold nanoparticles. The nanoparticles were characterized by transmission electron microscopy (TEM), and the diffraction peaks of GO and gold nanoparticles were confirmed through X-ray diffraction (XRD) analysis. Additionally, the optical absorbance of the nanoparticles was measured in the range of 200 - 900 nm using UV-Visible spectroscopy. A plasma generator was fabricated to produce cold plasma using helium (He) and oxygen (O₂) gases at a 99:1 ratio. The radicals generated by the cold plasma were analyzed via optical emission spectroscopy (OES). Cell treatment was conducted by applying various concentrations of GO and GO/Au nanoparticles. Cellular phenotype was monitored through optical microscopy, and biocompatible concentrations of both nanoparticles were determined using the 3-[4,5-dimethylthiazol-2-yl]-2,5 diphenyl tetrazolium bromide (MTT) assay. Subsequently, cold plasma at varying distances and durations was applied to the nanoparticle-treated cells. The generated radicals and the expression of apoptotic genes in treated cells were assessed using 2,2-diphenyl-1-picrylhydrazyl (DPPH) and real-time PCR, respectively.

**Results:**

The width of the bacillus-like gold nanoparticles was 15.13 ± 0.96 nm. The cold plasma generated radicals such as N2I2⁺, N2II1⁻, He•, and O⁻•. XRD analysis confirmed the successful coupling of gold onto the GO nanosheets. The biocompatible concentrations of GO and GO/Au nanoparticles were found to be 30 µg/100 µL and 20 µg/100 µL, respectively, as determined by the MTT assay. Radical formation increased as incubation time was extended from 30 to 60 seconds. Furthermore, real-time PCR analysis demonstrated the highest levels of p53, Bax, and caspase 3/8 expression at a plasma exposure time of 60 seconds in the composite-treated group, while Bcl2 expression was significantly reduced.

**Conclusions:**

The findings suggest that the parameters of heliox cold plasma and the concentrations of GO/Au nanoparticles must be optimized to effectively induce apoptosis in cervical cancer cells.

## 1. Background

Cervical cancer ranks fourth in terms of mortality among cancers globally, with approximately 90% of cases occurring in developing countries (1). A novel approach to generating reactive oxygen species (ROS) and reactive nitrogen species (RNS) is cold atmospheric plasma (CAP). These reactive chemical compounds play key roles in microbial destruction by macrophages (2), vasodilation (3), maintaining homeostasis (4), immune system responses (5), and regulating cell proliferation (6) and differentiation (7). Cold atmospheric plasma has the potential to selectively target and kill cancer cells, distinguishing it from traditional cancer therapies (8). Elevated levels of ROS can effectively damage cancer cells, while normal cells are more capable of tolerating these high concentrations of radicals. As a result, ROS and RNS can selectively destroy cancer cells depending on their physiological state (9).

Cold plasma has emerged as a promising therapeutic option, particularly for cancers that demonstrate resistance to conventional anticancer drugs, such as chondrosarcoma (10). Previous studies have shown that CAP induces apoptosis in approximately 46% of multidrug-resistant malignant cells (11). The CAP-induced cell death pathway is primarily linked to oxidative stress, which leads to mitochondrial and endoplasmic reticulum dysfunction and ultimately results in cell death (12).

In contrast to traditional cancer therapies like chemotherapy, radiation, and surgery, which often cause significant side effects and damage to surrounding healthy tissues (13), CAP offers a non-invasive alternative with minimal damage to healthy cells and tissues. Additionally, cancer drugs are often costly and associated with pain and other side effects, while CAP presents a more cost-effective and less harmful therapeutic option (14). Compared to ultraviolet (UV) radiation and conventional cancer drugs, CAP has shown better efficacy, as a study confirmed that UV photons have no effect on melanoma cancer cells (B16/F10) (15). The apoptotic effects of cold plasma on cancer cells were first reported by Fridman et al. in 2007 (16).

In relation to tumor cell transformation, gold nanoparticles (AuNPs) have been shown to strongly activate the PI3K/AKT signaling pathway in the presence of cold plasma. This combination therapy also reverses epithelial-mesenchymal transition (EMT) by increasing the expression of epithelial markers (17). In another study, AuNPs bound to phosphorylated FAK (p-FAK) selectively induced cell death in oral squamous cell carcinoma (OSCC) when combined with cold plasma. The apoptotic effect of the nanoparticles and cold plasma was significantly diminished when used separately, highlighting the synergistic nature of the treatment (18).

Similarly, magnetic nanoparticles loaded with paclitaxel and combined with cold plasma were applied to A549 cells, resulting in reduced drug resistance in these cells (19). Further research demonstrated that the combination of magnetic nanoparticles and non-thermal plasma decreased the expression of epidermal growth factor receptor (EGFR) (20). Moreover, a study investigated the synergistic effects of various nanoparticles, including silica, silver, iron oxide, cerium oxide, titanium oxide, and iron-doped titanium oxide, when combined with cold plasma on melanoma cell lines. The results showed an enhanced anticancer effect of these nanoparticles, though their efficacy varied depending on the nanoparticle and cell line types (21).

Iron magnetic nanoparticles were also used to evaluate the expression of the Bax/Bcl2 ratio, which favors apoptosis. The combination of these nanoparticles with cold plasma exhibited higher cytotoxicity (22). Silver nanoparticles have also been tested in conjunction with cold plasma, demonstrating a 100-fold increase in cytotoxicity against glioblastoma cells, which was ROS-dependent and mitigated by the addition of N-Acetyl Cysteine (23).

Additionally, Ag/TiO_2_-reduced graphene oxide (rGO) was employed for water purification, but the results indicated that while viruses were successfully destroyed, bacteria remained unaffected (24). Cerium oxide nanoparticles have also been studied for their protective role in the presence of cold plasma, particularly in safeguarding primary embryonic mouse fibroblasts (25).

In a similar study, platinum nanoparticles were found to reduce the production of radical species in U-251 MG cells when CAP was applied (26). Additionally, silica nanoparticles loaded with doxorubicin were combined with cold plasma in the treatment of the MCF-7 cell line, resulting in higher levels of apoptosis compared to the use of cold plasma alone (27). Silicon dioxide nanoparticles, another derivative of silica, have also been employed to promote growth in Astragalus fridae when used in conjunction with cold plasma (28).

Among various materials, AuNPs have gained attention for their use in photothermal applications due to their surface plasmon resonance (SPR), strong scattering properties, and biocompatibility. In fact, AuNPs have been approved by the U.S. Food and Drug Administration (FDA) for such purposes (29). One study demonstrated that AuNPs combined with CAP were more effective in killing OSCC cells than CAP treatment alone (18). However, the efficacy of these particles is highly dependent on their size, and their photothermal properties are significantly diminished in the absence of CAP (30). When biological molecules, such as proteins, enzymes, biological markers, drugs, or antibodies are conjugated with AuNPs, they serve as highly efficient markers for detecting bacteria and cancer cells, as well as delivering conjugated drugs to tumor sites (31).

Due to the enhanced permeability and retention (EPR) effect, nanoparticles tend to accumulate in tumors with leaky vascular systems, a characteristic feature of solid tumors (32). Transcytosis, a process by which nanoparticles are transported from the bloodstream through the tight junctions of endothelial cells into tumor tissue, is another pathway utilized for delivering nanoparticles to tumors (33).

Given the challenges posed by renal and hepatic clearance of nanoparticles, it is more advantageous to load them into carriers with extended circulation times, such as graphene derivatives. Graphene sheets, known for their strong photothermal effects due to the large number of π-π conjugations between the sheets, are particularly suitable for this purpose (34). Moreover, graphene platelets are highly efficient at conducting heat to loaded nanoparticles, such as MnO_2_, further enhancing their therapeutic potential (35).

In this context, the low quantum yield of graphene particles can be enhanced, improving the conversion of radiation into heat. When plasma irradiation is applied, the free electrons in graphene nanoparticles begin to oscillate, generating heat that is transferred to the surface where the AuNPs are doped. Cold atmospheric plasma not only generates reactive chemical species but also facilitates cell penetration by creating a strong electric field, similar to electroporation, allowing large molecules to cross the cell membrane more easily (36). In one study, a graphene/gold nanohybrid was produced, modified with folate and paclitaxel, and resulted in a high rate of cancer cell death due to hyperthermia and laser irradiation (37). Another investigation evaluated the efficiency of near-infrared (NIR) light excitation and graphene/gold nanoparticles in human glioblastoma astrocytoma cells, showing significant tumor suppression (38).

Furthermore, this nanocomposite limited cell viability in human cervical cancer (HeLa) cells based on concentration and promoted the expression of apoptotic and autophagic genes, along with higher levels of ROS (39). A separate study confirmed that the cytotoxicity of AuNPs was 25 times more potent when combined with cold plasma on the glioblastoma multiforme cell line, primarily due to enhanced endocytosis caused by the synergistic effect of the nanoparticles and plasma. The nanoparticles accumulated more effectively in the lysosomes of cells treated with both nanoparticles and plasma, increasing apoptosis without disrupting the transient physical integrity of cellular membranes (40).

Another experiment investigated the effect of combining AuNPs with cold plasma on mice with breast cancer, showing rapid tumor inhibition during early treatment stages. However, the in vitro results on the L929 cell line were less satisfactory (41). In a separate study, AuNPs conjugated with antibodies were tested with air-cold plasma on melanoma cells, revealing a positive effect on the rate of cell death. The proposed mechanism suggested that the accumulation of nanoparticles inside cells led to increased toxicity, even at low doses of cold plasma (42).

Regarding glioblastoma, combining AuNPs with cold plasma resulted in heightened ROS production, causing oxidative stress that disrupted intracellular signaling pathways and damaged proteins, lipids, and DNA (43). In a study targeting colorectal cancer, AuNPs were combined with cold plasma, showing a significant impact on cell death. Helium (He) was used to generate the plasma, and oxygen (O₂) was added to increase ROS levels (29).

In some studies, nanoparticles have been modified with growth factors, such as the epidermal growth factor (EGF), which is overexpressed in cancer cells like lung carcinoma cells. These modifications resulted in increased cell apoptosis after applying the plasma treatment (44). However, there are no reported studies combining AuNPs doped on graphene oxide (GO) sheets with cold plasma for cancer treatment. Furthermore, the application of this strategy in cervical cancer has not yet been explored as a potential therapeutic method. In this study, GO nanosheets were combined with AuNPs, and a He/O₂ plasma jet was developed. The HeLa cell line was cultured in the presence of these nanoparticles, and the CAP parameters, including time and distance, were optimized. The study further examined cell viability, gene expression, and the radicals generated by treated cells.

## 2. Objectives

**Figure 1.**
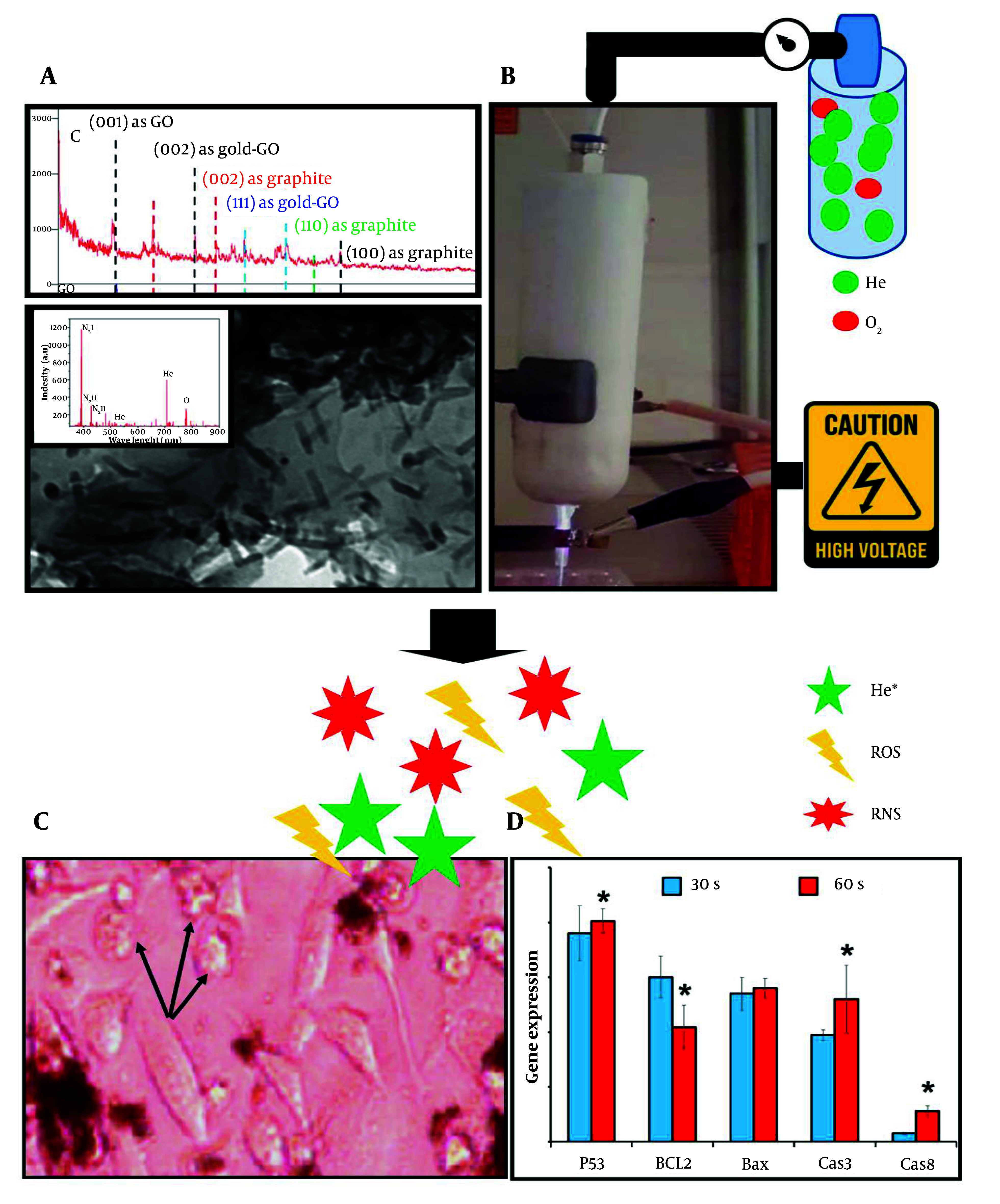
The schematic graphical abstract illustrates the use of helium (He) and oxygen (O₂) gases at a 99:1 ratio to generate cold plasma in combination with graphene oxide (GO) nanoparticles doped with gold. The nanoparticles were synthesized and characterized by XRD, optical emission spectroscopy (OES), and TEM. After confirming their properties, biocompatible concentrations of 30 µg/100 µL for non-doped GO and 20 µg/100 µL for doped GO were established. The cells were then treated with these nanoparticles and exposed to stable cold plasma, generated with a voltage of 6 kV, frequency of 35 kHz, and power of 12 W. These parameters were consistent across all assessments. Cellular morphology was observed via optical microscopy, and apoptotic gene expression was analyzed using real-time PCR. The presence of round cell shapes, as opposed to normal polygonal forms, indicated apoptosis. Moreover, molecular analysis revealed the highest apoptotic gene expression when plasma treatment was applied for 60 seconds at a distance of 3 cm.

The main objective of this study was to optimize CAP parameters and nanoparticle concentrations to induce cell apoptosis. A schematic overview is provided in Figure 1. 

## 3. Methods

### 3.1. Fabrication of Cold Plasma Generator

**Figure 2.**
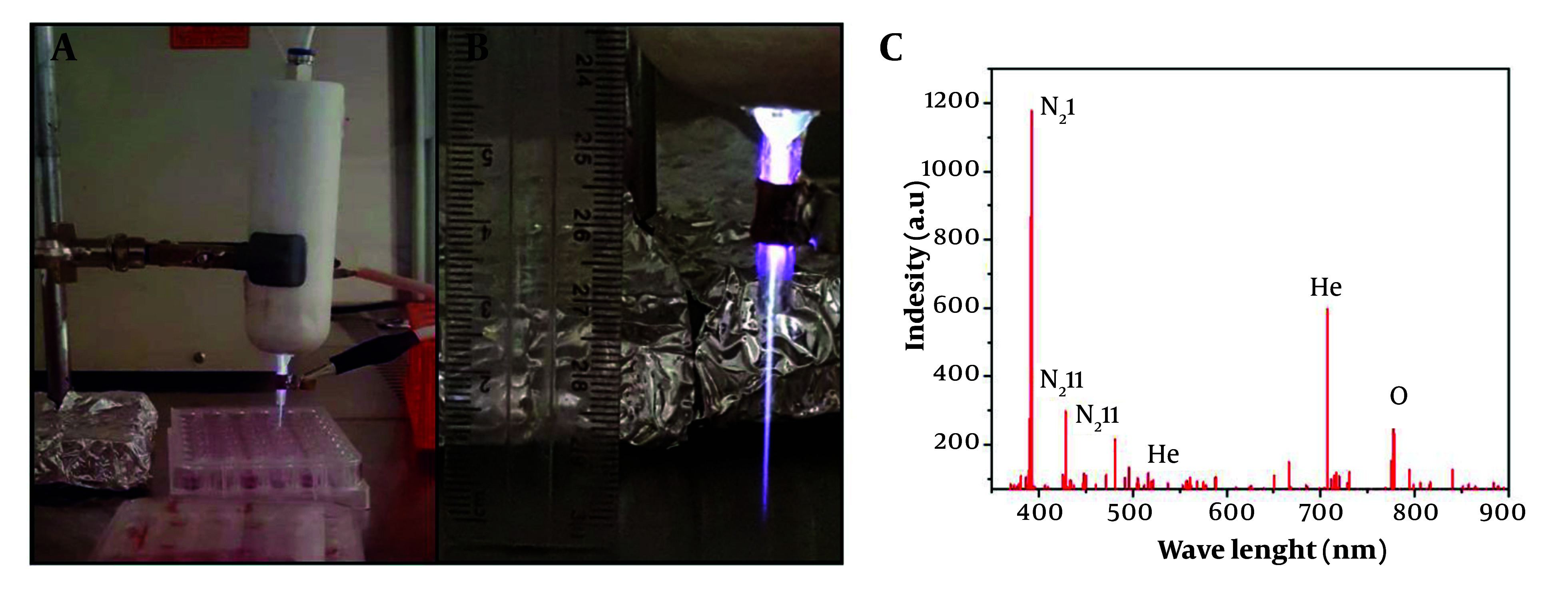
A and B, the helium (He) and oxygen (O₂) plasma jet setup, showing its placement at a 3 cm distance from the treated surface; C, the optical emission spectroscopy (OES) results displaying the generated radicals from the He and O₂ plasma jet.

An atmospheric plasma jet (High Tech Company, 0045, Tehran, Iran) filled with a mixture of He and O₂ gases at a 99:1 ratio, commonly referred to as "Heliox," was developed for this study. The plasma generator was equipped with two parallel cylindrical copper electrodes: A conical-shaped copper cathode and a cylindrical outer anode with eight copper rods. The height and diameter of the electrodes were 45 mm and 0.12 mm, respectively. A stable plasma jet was produced under a high AC power supply with a voltage of 6 kV, frequency of 35 kHz, and power output of 12 W. These voltage and frequency values were achieved using a transformer (Phenix Technologies, MD, USA). Initial plasma formation was conducted with default values, which were later adjusted to stabilize the irradiations, as depicted in Figure 2A (45).

### 3.2. Measurement of Radical Species

Optical emission spectroscopy (OES) (TIDA Spectrometer 350 - 1100, Teksan, Tehran, Iran) was employed to analyze the UV-visible emission spectra related to the radicals generated by the cold plasma. The spectrometer operated with a resolution of 0.02 nm and a power of 200 mA at +5 VDC, covering a wavelength range between 300 - 900 nm. The emitted light from the plasma's radicals was captured and measured by the spectrometer (46).

### 3.3. Graphene Oxide Fabrication

The well-known Hummer’s method was used to produce GOe nanoparticles. In this process, graphite (Sigma-Aldrich) was combined with 0.5 g of sulfuric acid (98%, Merck). The suspension was stirred at a temperature below 0°C for 10 minutes. Sodium nitrite (0.5 gr, Sigma) was then added to the suspension, followed by 3 gr of potassium permanganate, and the temperature was raised to 30°C. The reaction was maintained for 4 hours, after which 100 mL of water was added. The temperature was again raised, this time to 90°C, and an additional 50 mL of water was added, along with 3 mL of hydrogen peroxide (Merck). After an ultra-sonication step, the solution was washed several times to adjust the pH to 7. Finally, the solution was dried at 50°C for 24 hours (47).

### 3.4. Gold Doping

An aqueous solution was prepared by mixing GO and AuNPs (US-Nano, USA) at respective concentrations of 0.3 mg/mL and 0.05 mg/mL in a 3:10 ratio. The mixture was heated to 80°C for 30 minutes, followed by the addition of sodium citrate as a chemical reducing agent. The solution was shaken continuously for 5 hours, and the resulting nanoparticles were collected by centrifugation at 6000 rpm. The collected sample was dried in an oven at 40°C to remove any remaining liquid. This method was adapted from previous studies with slight modifications (48).

### 3.5. Transmission Electron Microscopy, XRD, and UV-Vis Spectroscopy

The doped and non-doped nanoparticles were analyzed for their size and homogeneity using transmission electron microscopy (TEM). For this analysis, the nanoparticles were fixed on carbon grids and examined using a TEM instrument (Philips CM-30) operating at an acceleration voltage of 250 kV. The successful incorporation of AuNPs into GO nanosheets was confirmed using X-ray diffraction (XRD, AXS Bruker diffractometer) with a CuKα radiation source, generating a wavelength of 1.5418 Å. Additionally, the optical properties of the doped and non-doped GO nanoparticles were evaluated using UV-Vis spectroscopy (V-670, JASCO, Japan) across the 200 - 900 nm wavelength range. A quartz cuvette with an optical path length of 1 cm was used for this analysis (49, 50).

### 3.6. MTT Assay

The human epithelial carcinoma (HeLa) cell line was obtained from the Pasteur Institute of Iran and cultured in high glucose Dulbecco’s Modified Eagle’s Medium (DMEM, Gibco), supplemented with 10% fetal bovine serum (FBS, Gibco) and 2 mM L-glutamine (Gibco). The cells were maintained at 37°C in a humidified incubator containing 5% CO_2_ and 20% O_2_ and passaged with trypsin (Gibco) when cell confluence exceeded 80%. Cell viability was assessed using the MTT assay. HeLa cells were seeded in 96-well plates at a density of 5 × 10^3^ cells per well and incubated for 24 hours. Subsequently, both composite (GO/Au) and non-composite GO nanoparticles were applied at serial concentrations of 5, 10, 15, 20, 25, and 30 µg/100 µL. Untreated cells served as the control group. After 24, 48, and 72 hours of incubation, the MTT solution (0.1%) was added to the wells, and after 3.5 hours, the formazan crystals were dissolved using dimethyl sulfoxide (DMSO, Merck). Absorbance was measured at 570 nm using a microplate reader (BioTek Instruments, USA) (51). Cell viability was calculated using the following formula:


$$Cell\;viability\%=\;\frac{OD\;of\;treated\;cells}{OD\;of\;TCPS\;\times 100}$$

Where OD refers to the optical density at 570 nm, and the control cells are untreated cells grown on tissue culture polystyrene (TCPS).

The concentration with a cell viability value greater than 90% was reported as the biocompatible value and used for subsequent cellular investigations in the presence of cold plasma. This assessment was repeated following the combination of the nanoparticles and plasma. This step was essential to identify the plasma conditions capable of killing at least 50% of the cells in a synergistic strategy with the nanoparticles. The time and distance parameters for plasma application were set to 0.5, 1, and 3 minutes at a distance of 3 cm, with an additional group treated for 1 minute at a distance of 5 cm.

### 3.7. DPPH Assay

The measurement of radicals generated in the treated cells by the nanoparticles and plasma was conducted using a DPPH assay. Plasma was applied to all groups for 30 and 60 seconds at a distance of 3 cm. Then, DPPH solution (Sigma, 0.1 mM) was added at 0.5 mL per well in a 24-well plate, using methanol (Merck) as the dissolution medium. The scavenging activity required at least 30 minutes at room temperature, after which the absorbance values of the samples were measured at 520 nm. The following formula was used to calculate the radical inhibition percentage:


$$Radical\;inhibition\;percentage=\;\frac{{OD}_{blank}-{OD}_{sample}}{{OD}_{blank}\times 100}$$

Here, the OD value of the blank represents the absorbance of the DPPH solution without any treatment, and the OD of the sample represents the absorbance of the treated groups (47).

### 3.8. Real-time PCR

For genetic analysis, HeLa cells were cultured at a density of 2 × 10^4^ cells per well in a 24-well plate. The experimental groups included the TCPS group (control) and nanoparticle-treated groups. All groups were exposed to plasma for 30 and 60 seconds at a distance of 3 cm. After 72 hours, the cells were harvested for RNA extraction, with the TCPS group serving as the control to normalize gene expression values. First, TRIzol reagent (Gibco) was used to extract total RNA. The quality of the extracted RNA was verified by running 5 μL of the RNA on a 1.5% agarose gel (Gibco) followed by electrophoresis. To ensure RNA purity, its absorbance ratio at 260/280 was measured after dilution with RNase-DNase-free water (Gibco). Subsequently, a cDNA synthesis kit (Yekta Tajhiz Azma, Tehran, Iran) was employed, and Random Hexamer primers (SinaClon, Iran, 1 μL) were mixed with 1 μg of RNA in 9 μL of RNase-DNase-free water. The cDNA samples (0.5 μL) were analyzed using the Corbett Rotor-Gene 6000 (QIAGEN Rotor-Gene Q, Germany) to assess the expression of the genes listed in Table 1. GAPDH was used as the reference gene for normalization of marker gene expression. PCR reactions were conducted with a SYBR Green master mix, and the primers were synthesized by Cinagen, Iran (52).

**Table 1.** Primer Sequences of Marker and Internal Control Genes for Real-time PCR

Gene	Sequence Forward	Sequence Reverse
**GAPDH**	GGTGAAGGTCGGAGTCAACG	GACAAGCTTCCCGTTCTCAGC
**Bax**	GCGTCCACCAAGAAGCTGAG	GATCAGTTCCGGCACCTTGG
**Bcl2**	GAGTTCGGTGGGGTCATGTG	GATAGGCACCCAGGGTGATG
**CAS 3**	CAGTCGCTTTGTGCCATGCTG	CCCTCTGCAGCATGAGAGTAG
**CAS 8**	TCTGGCCTCCCTCAAGTTCC	TTTGAGCCCTGCCTGGTGTC
**p53**	CTAAGCGAGCACTGCCCAAC	ATGGCGGGAGGTAGACTGAC

### 3.9. Statistical Analysis

All experiments were conducted at least in triplicate, except for the XRD analysis. Sigma-Plot software was used for statistical analyses. A Student’s *t*-test was applied to compare two groups, while one-way ANOVA was used for comparisons involving more than two groups. Results were presented as mean ± standard deviation (SD), with P-values ≤ 0.05 considered statistically significant.

## 4. Results

### 4.1. Optical Emission Spectroscopy Characterization of Radical Species

A detector tube was used to measure the generated radicals, and the results are shown in Figure 2B. The dominant reactive species were detected in the UV/VIS range of 300 - 900 nm. According to the curve, most of the N₂ and He spectra are observed in the 380 - 550 nm range, while the O₂ species are associated with wavelengths between 700 - 800 nm. Based on previous studies, the N₂ bonds between 380 - 450 nm are due to neutral and ionic emission vibrations of nitrogen molecules (53). The peak at 375 nm corresponds to the second positive system of N₂ (N₂ I, 2+) (54), while other nitrogen peaks are attributed to the first negative system of N₂ (N₂ II, 1-) at 405 nm (55) and 427 nm (56). For He, strong peaks were detected at 587 nm (57) and 706 nm (58). For atomic O₂ groups, a sharp peak appears at 777 nm (59). Additional specific peaks of O₂ radicals were observed at 844 nm and 760 nm, respectively (60). The latter band is associated with the O-O magnetic dipole transitions of O₂ (61). Additionally, some smaller peaks around 725 nm are related to atomic O₂ (62).

### 4.2. Transmission Electron Microscopy, XRD, and UV-Vis Spectroscopy of Nanoparticles

**Figure 3.**
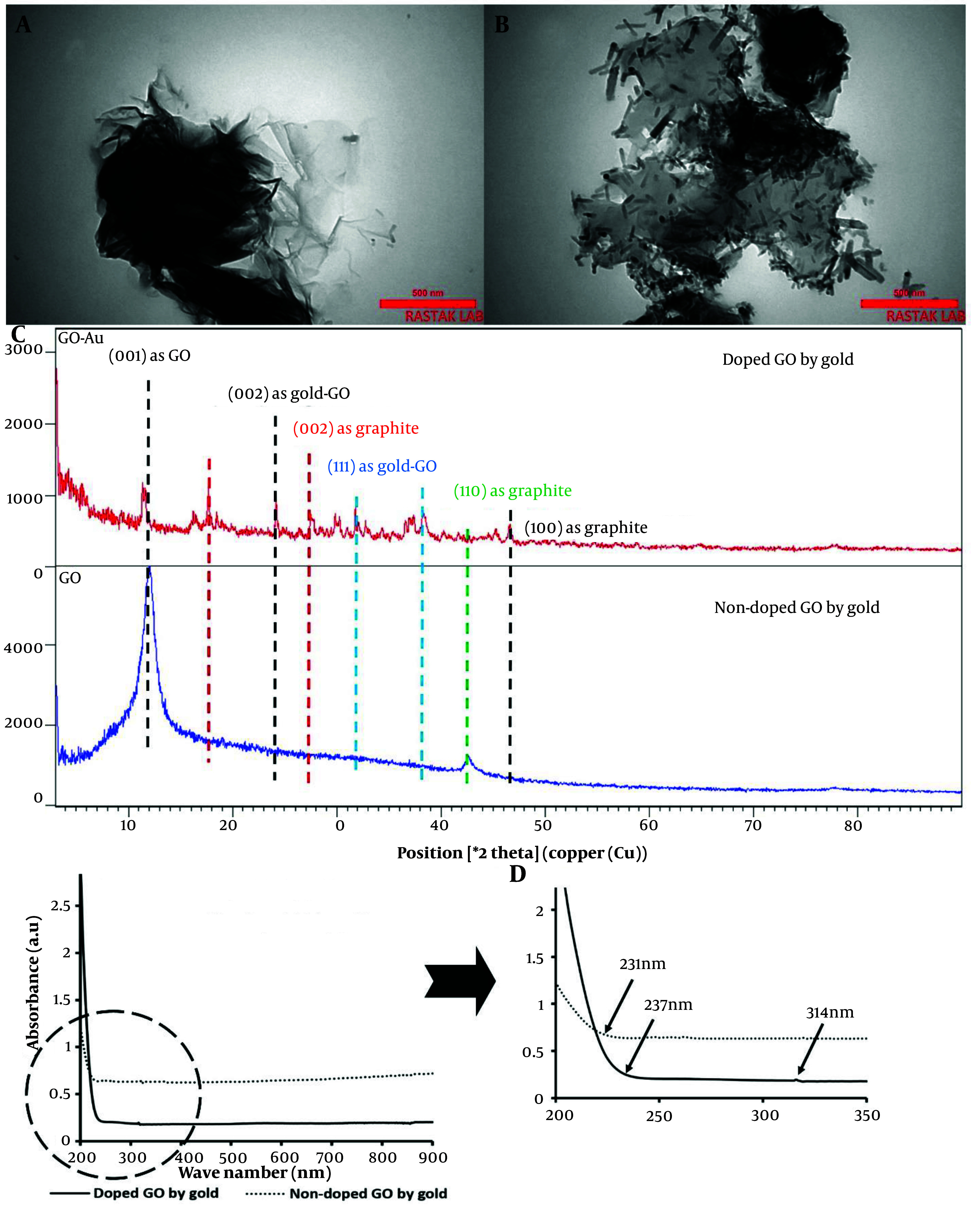
A, transmission electron microscopy (TEM) image of graphene oxide (GO) nanosheets; B, TEM image of GO nanosheets doped with gold nanoparticles; C, XRD analysis of GO nanosheets, comparing doped and non-doped samples with gold nanorods; D, Absorbance values of doped and non-doped GO with gold across the wavelength range of 200 - 900 nm, with a detailed view of the 200 - 350 nm region.

The TEM results are presented in Figure 3A and B, showing the undoped and doped nanoparticles. The images confirmed the sheet-like structure of the GO nanosheets, which displayed clear transparency. In contrast, the composite type exhibited rod-shaped AuNPs. Upon doping the GO nanosheets with AuNPs, bacillus-like particles with an average length of 77.35 ± 22 nm and a width of 15.13 ± 0.96 nm were deposited on the graphene nanoplatelets. These particles were evenly distributed across all GO nanosheets.

The nanoparticles, both coupled and non-coupled types, were evaluated using XRD, and their patterns confirmed the presence of the following peaks (Figure 3C). For the undecorated nanosheets, a peak at 2θ = 12° was assigned to the (001) plane. Additionally, a significant peak at 2θ = 18° with the (002) plane indicates the graphitic structure of graphene, where the spacing between graphene planes is small (63). The peak at 2θ = 24° represented the reduction of GO nanoparticles with gold after their implantation on the sheets (64), corresponding to the (002) plane of gold. Furthermore, the diffraction peak at 2θ = 32° is another gold nanoparticle peak that appeared on the GO layers (65), corresponding to the (111) plane. A similar peak at 2θ = 38°, associated with the (111) plane of AuNPs, was also prominent (66). Another peak at a diffraction angle of 27° corresponded to the graphite reflection of the (002) plane (67). Additionally, a bond at 2θ = 43° was associated with the graphite of GO at the (110) plane (68). A small band with the doped GO at 47° was detected, representing graphite with the (100) plane (69).

The optical properties of GO nanoparticles, both doped and non-doped with gold, were displayed in Figure 3D. GO nanosheets showed a broadening shoulder with maximum absorbance between 200 - 231 nm, corresponding to the π – π* transitions of aromatic C=C bonds (70). A similar band, though with a higher value, was detected for the doped nanoparticles, extending from 200 to 237 nm. Additionally, the n → π* transitions of the C=O bonds at 300 nm in GO nanoparticles were redshifted to 314 nm due to the reduced form of these nanoparticles when doped with gold (71).

### 4.3. Cell Phenotype

**Figure 4.**
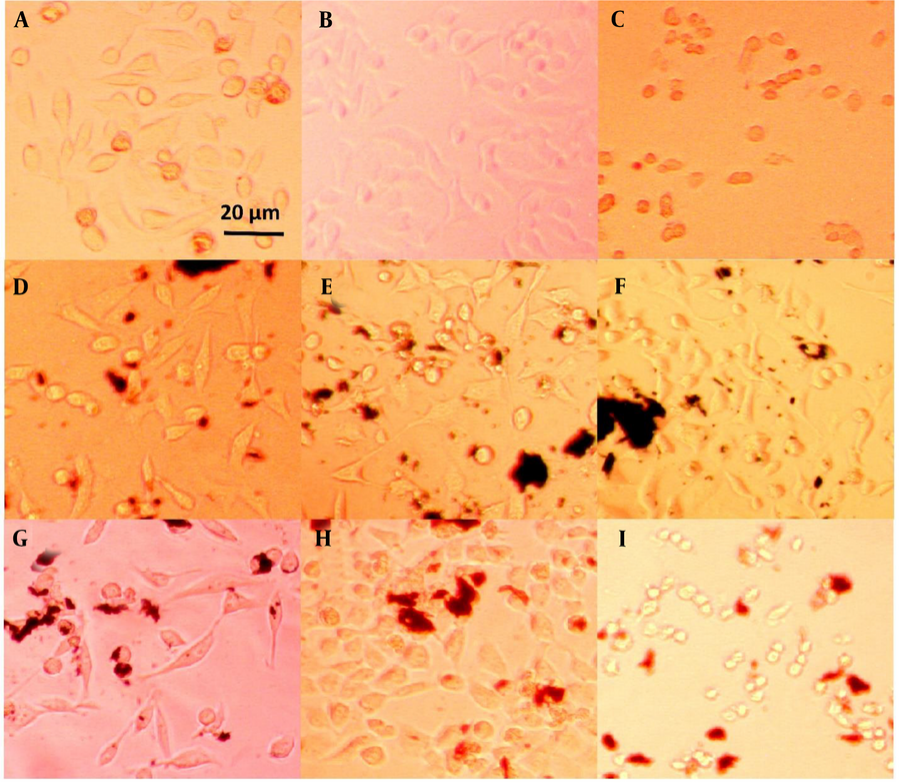
HeLa cell line images after 24 hours of treatment: A, plasma only for 1 min at 5 cm; B, plasma only for 1 min at 3 cm; C, plasma only for 3 min at 3 cm; D, non-doped particles for 1 min at 5 cm; E, non-doped particles for 1 min at 3 cm; F, non-doped particles for 3 min at 3 cm; G, doped particles for 1 min at 5 cm; H, doped particles for 1 min at 3 cm; I, doped particles for 3 min at 3 cm.

**Figure 5.**
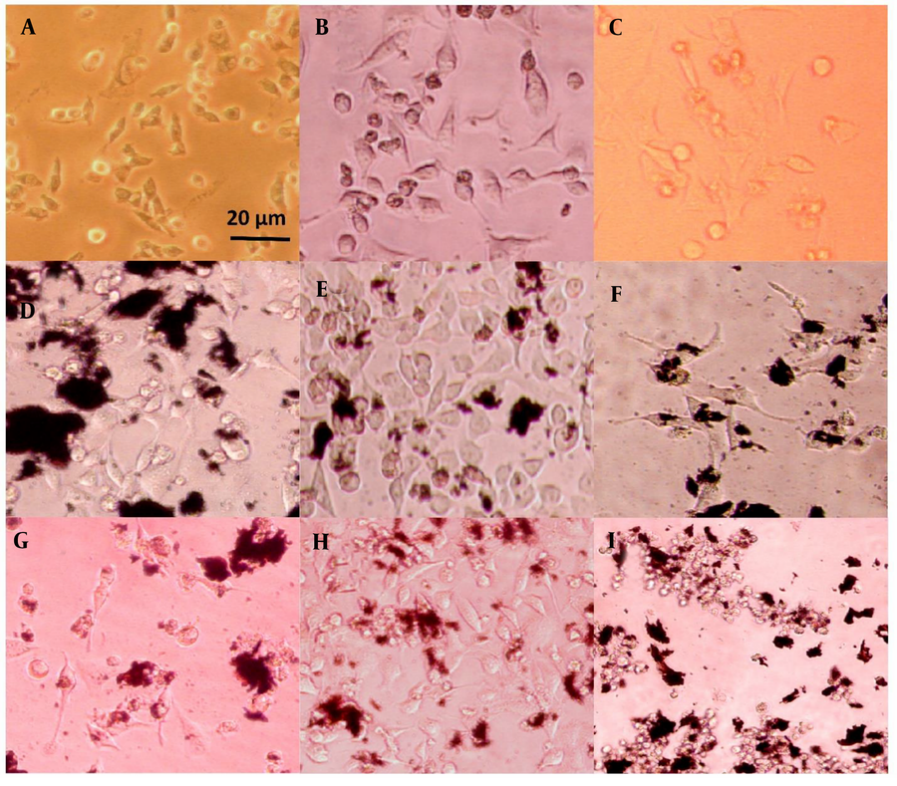
HeLa cell line images after 72 hours of treatment: A, plasma only for 1 min at 5 cm, B, plasma only for 1 min at 3 cm; C, plasma only for 3 min at 3 cm; D, non-doped particles for 1 min at 5 cm; E, non-doped particles for 1 min at 3 cm; F, non-doped particles for 3 min at 3 cm; G, doped particles for 1 min at 5 cm; H, doped particles for 1 min at 3 cm; I, doped particles for 3 min at 3 cm.

The morphology of plasma-treated cells was observed under a light microscope after 24 and 72 hours, as shown in Figures 4, and 5, respectively. Among the groups without nanoparticles, the group treated with plasma at a distance of 5 cm for 1 minute exhibited a higher cell count compared to the group treated at a distance of 3 cm. In contrast, the group treated for 3 minutes at a distance of 3 cm showed only a few single cells, as opposed to the other two groups, which had visible cell colonies. Previous studies have shown that the distance between the plasma nozzle and the treated surface significantly impacts the formation of radical species, with higher radical concentrations achieved at shorter distances (72).

On the other hand, the groups containing non-doped GO nanoparticles displayed no noticeable difference in cell morphology. This suggests that the effect of these nanoparticles on cell numbers cannot be detected using an optical microscope and requires more sensitive methods, such as MTT analysis. The transformation of cells from a regular polygonal shape to a rounded form indicates the initiation of apoptosis (73).

A significant morphological change was observed in the groups treated with the coupled nanoparticles (GO/Au). The cells exhibited a collapsed structure and lost their adhesion properties. Furthermore, when the plasma jet distance was reduced to 3 cm, the number of attached cells increased, even with a treatment duration of 3 minutes. This highlights the importance of plasma parameters, such as distance and time, in influencing cell viability and attachment.

### 4.4. MTT Assessment

**Figure 6.**
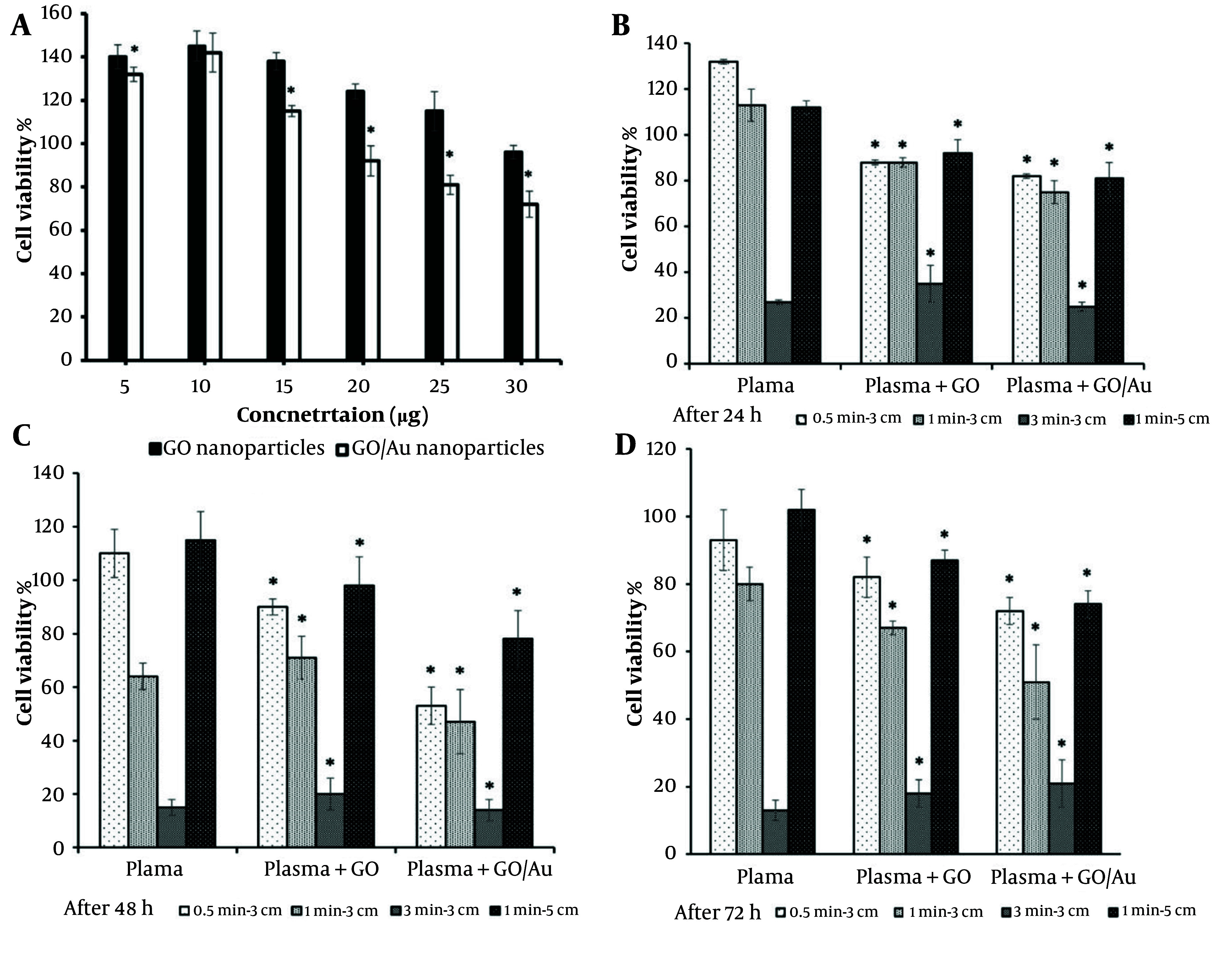
MTT results: A, a gradient of particles in the absence of plasma: Comparisons between two cell groups treated with graphene oxide (GO) (control group) and GO/Au (test group) nanoparticles; B, after 24 hours of treatment with 30 µg/100 µL of non-doped GO and 20 µg/100 µL of doped GO nanoparticles: Comparisons between the cell groups treated with only cold plasma (control) and those treated with both cold plasma and nanoparticles (test groups); C, after 48 hours of treatment with 30 µg/100 µL of non-doped GO and 20 µg/100 µL of doped GO nanoparticles: Comparisons between the cell groups treated with only cold plasma (control) and those treated with both cold plasma and nanoparticles (test groups); D, after 72 hours of treatment with 30 µg/100 µL of non-doped GO and 20 µg/100 µL of doped GO nanoparticles: Comparisons between the cell groups treated with only cold plasma (control) and those treated with both cold plasma and nanoparticles (test groups). The star (*) indicates statistical differences when P-value ≤ 0.05.

The results of the serial concentration tests of the nanoparticles showed that the biocompatible concentrations of GO and GO/Au nanoparticles were 30 and 20 µg/100 µL, respectively, with cell viability percentages of 96 ± 3.1% and 92 ± 4.5% (Figure 6A). These values fall under the first-grade cytotoxicity classification, as reported by previous studies (74). A similar study observed comparable values for AuNPs at concentrations of 375 and 187 ppm in HCT-116 cells (29). These optimized concentrations were used to treat the cells, and the subsequent effects of plasma treatments were then assessed.

As shown in Figures 6B - D, cell viability decreased significantly with increasing plasma treatment time (P ≤ 0.05). In both groups—those treated with nanoparticles and those without—the cell viability dropped to less than 40%. In groups treated for 0.5 and 1 minute, the effect of the nanoparticles was noticeable. The 0.5-minute treatment group showed cell viability above 50%, indicating that this condition was insufficient to reach the IC_50_ threshold. However, there was a strong correlation between the groups treated with plasma alone and those treated with both nanoparticles and plasma (P ≤ 0.05). Furthermore, the differences between the GO and GO/Au groups were significant at all time points (P < 0.05).

For the composite nanoparticle group, cell viability decreased to 75%, 47%, and 51% at 24, 48, and 72 hours, respectively, when the plasma treatment condition was set to 1 minute at a distance of 3 cm. Under the same plasma treatment conditions, the non-doped nanoparticle group showed cell viability of 88%, 71%, and 67% at the corresponding time points. After 72 hours, cell viability gradually decreased to 67% and 51% in the group treated with plasma for 1 minute at 3 cm. When the treatment time was extended to 3 minutes at the same distance, the differences in cell viability between the groups became statistically insignificant (P > 0.05), indicating the substantial impact of 3 minutes of plasma exposure.

Increasing the plasma nozzle distance from 3 to 5 cm resulted in cell viabilities similar to those observed with 30 seconds of plasma treatment. Overall, both the treatment duration and the position of the plasma jet significantly influenced the optical activity of the nanoparticles. The 1 minute at 3 cm plasma treatment condition was identified as the optimal setting to reduce cell viability by approximately 50%, which was used for the subsequent assays.

### 4.5. DPPH Radical Scavenging

**Figure 7.**
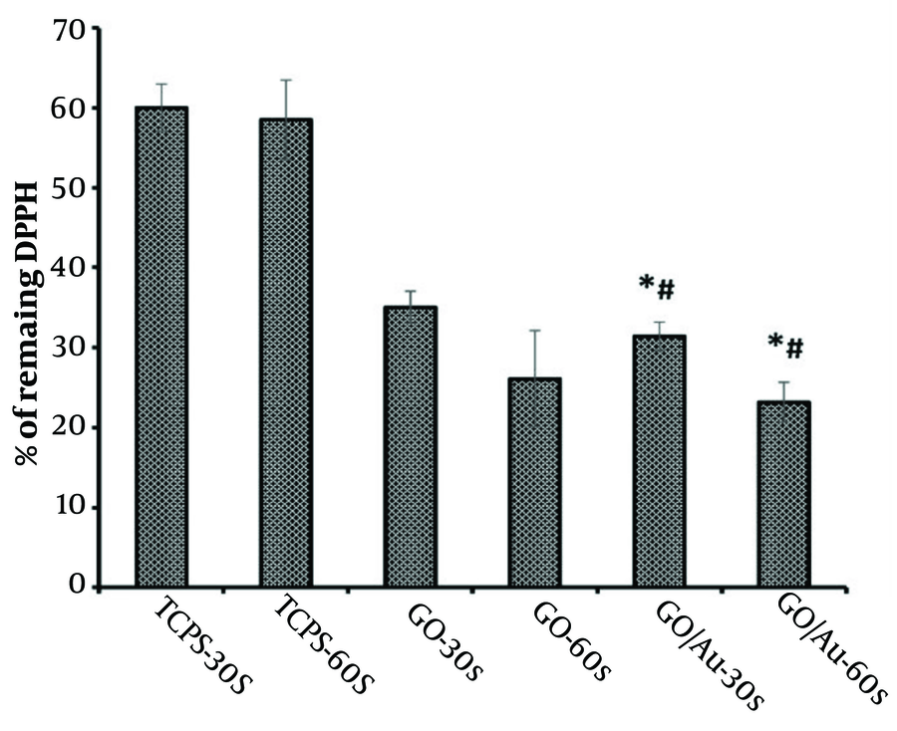
The DPPH scavenging curve (%) showing absorbance values at 520 nm after treatment of HeLa cells with 30 µg/100 µL of non-doped graphene oxide (GO)and 20 µg/100 µL of doped GO nanoparticles. The asterisk (*) indicates statistical differences between the doped group and TCPS, and the number sign (#) indicates statistical differences between the doped and non-doped groups when P-value ≤ 0.05.

The anticancer properties of CAP are largely due to its ability to generate reactive radical species (75). The quantity of these generated radicals was assessed using the DPPH method, as depicted in Figure 7. The results showed that the TCPS groups had relatively consistent values (P > 0.05) and exhibited higher absorbance than the other groups. This outcome confirms the synergistic effect of the nanoparticles and plasma in producing radical species.

The amount of generated radicals increased after treating the cells with the nanoparticles, both GO and gold. This effect was further enhanced following plasma treatment in both nanoparticle groups. Specifically, the radical generation of the bare nanoparticles shifted from 35 ± 2% to 23.15 ± 6% as the plasma treatment duration increased from 30 to 60 seconds. Similarly, the radical generation for the coupled nanoparticles decreased from 33.42 ± 1.8% to 26.25 ± 2.5% after 60 seconds of plasma exposure. There were negligible differences between the bare and composite nanoparticle groups when comparing the same incubation times (P > 0.05). However, there was a significant difference between the 30 and 60-second treatment durations, underscoring the role of plasma exposure time in radical synthesis (P ≤ 0.05).

### 4.6. Real-time PCR

**Figure 8.**
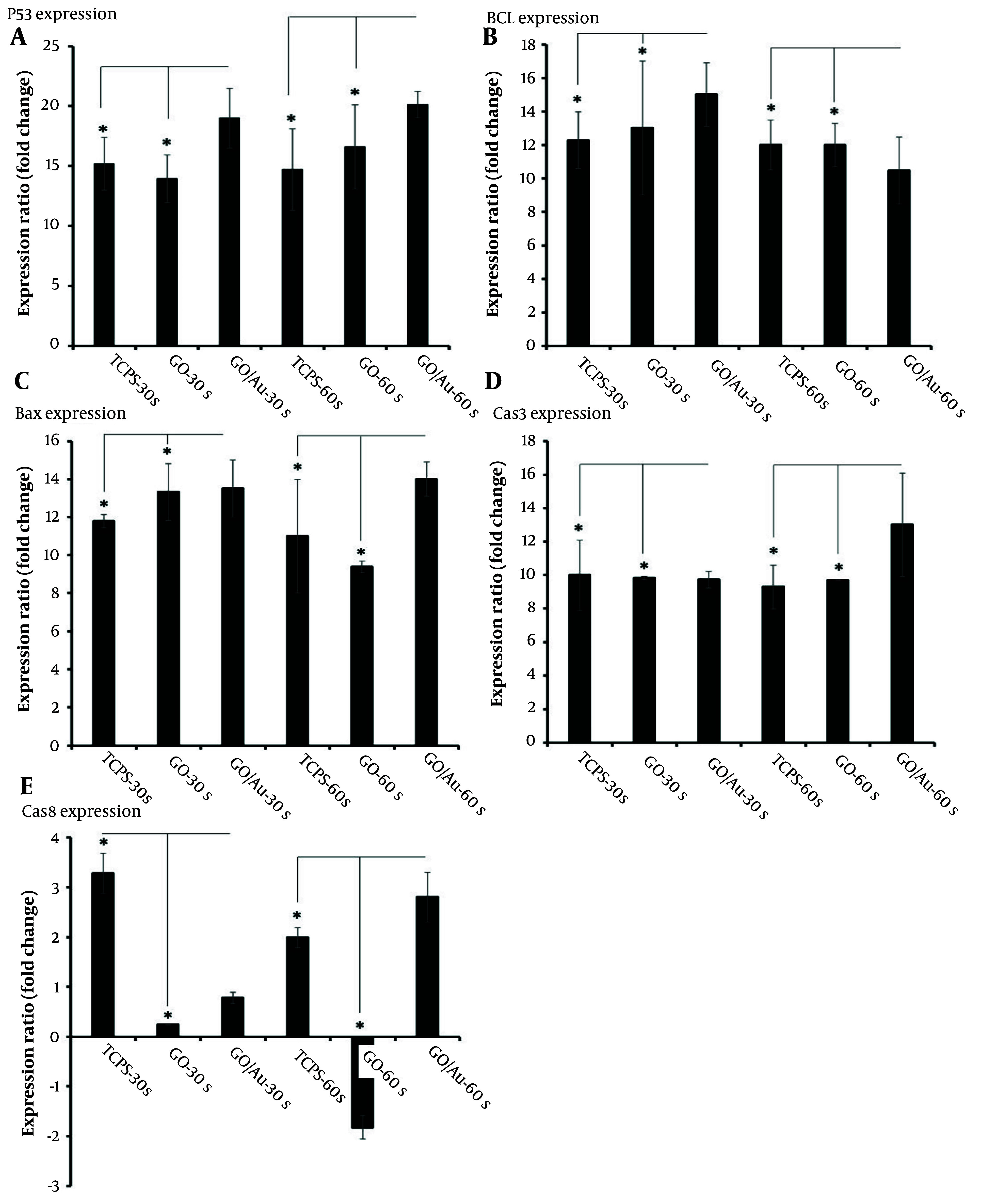
Real-time PCR analysis of HeLa cells after 72 hours of treatment with 30 µg/100 µL of non-doped graphene oxide (GO) and 20 µg/100 µL of doped GO nanoparticles. The star (*) indicates statistical differences between the TCPS and nanoparticle-treated groups with the same cold plasma treatment time when P-value ≤ 0.05. Statistical differences among all groups are provided in Appendix 1.

The expression of apoptosis-related genes can provide insights into the effects of plasma treatment on cell fate. In this study, apoptosis genes such as Bax, Bcl2, p53 (76), caspase 3, and caspase 8 (77) were evaluated to determine the molecular response of the cells. The results are shown in Figure 8. All gene expression values were normalized against the untreated TCPS group. The housekeeping gene GAPDH was used as a reference for calibrating the expression levels of the target genes. After 72 h, total RNA was extracted and used to detect the gene expression profile. Upon careful examination of specific apoptosis genes, Bcl2 was identified as an inhibitory gene for cell proliferation, thereby reducing cell maintenance (78).

The well-known Hummer’s method was used to produce GOe nanoparticles. In this process, graphite (Sigma-Aldrich) was combined with 0.5 g of sulfuric acid (98%, Merck). The suspension was stirred at a temperature below 0°C for 10 minutes. Sodium nitrite (0.5 gr, Sigma) was then added to the suspension, followed by 3 gr of potassium permanganate, and the temperature was raised to 30°C. The reaction was maintained for 4 hours, after which 100 mL of water was added. The temperature was again raised, this time to 90°C, and an additional 50 mL of water was added, along with 3 ml of hydrogen peroxide (Merck). After an ultra-sonication step, the solution was washed several times to adjust the pH to 7. Finally, the solution was dried at 50°C for 24 hours (47). In contrast, the groups treated for 30 seconds showed higher expression of Bcl2, though the difference between the composite and non-composite nanoparticle groups was not statistically significant (P > 0.05).

Overall, considering the expression levels of Bcl2 across the groups, it can be concluded that the role of composite nanoparticles becomes more prominent when the plasma treatment duration is extended. As mentioned earlier, untreated cells were used as the baseline for calibrating the other gene expressions. The higher Bcl2 expression observed in some groups may be attributed to uncontrolled cell proliferation. However, this increased cell proliferation may eventually lead to a reduction in the cell population as overcrowded cells begin to undergo self-destruction.

Another important gene, Bax, is associated with cell apoptosis (79). The group treated with composite nanoparticles and plasma for 60 seconds showed the highest Bax expression compared to other groups (P ≤ 0.05). In contrast, the group treated with GO nanoparticles and plasma for 30 seconds exhibited no significant difference when compared to the doped nanoparticles treated for the same duration. This suggests that even the bare nanoparticles could induce apoptosis, without the need for doping. This conclusion was further supported by the comparison between the TCPS and GO nanoparticle-treated groups under the same plasma conditions, where Bax expression was significantly higher in the nanoparticle-treated group (P ≤ 0.05).

Another gene, p53, known as an anti-survival gene (80), plays a critical role in cancer inhibition. The fold change in p53 expression for the composite nanoparticle group treated for 60 seconds was 20.13 ± 1.1, while the same group treated for 30 seconds had a fold change of 19 ± 2.5. There was no significant difference between these two groups and the others (P > 0.05), indicating that plasma treatment effectively induces p53 expression regardless of the duration.

Additionally, the expression of the apoptotic genes caspase 3 and caspase 8 further confirmed the anticancer efficacy of the CAP therapy. The highest expression levels of these genes were observed in the composite nanoparticle group treated with plasma for 60 seconds. Notably, caspase 3 expression was elevated, likely due to its activation by caspase 8 (81).

## 5. Discussion

Transmission electron microscopy results confirmed the presence of bacillus-like AuNPs with dimensions of 77.35 ± 22 nm in length and 15.13 ± 0.96 nm in width. These nanorods were uniformly distributed across the GO nanosheets. As consistent with other reports, these rod-shaped particles exhibit two SPR modes, including transverse and longitudinal types (82). The high aspect ratio, due to their small diameter, causes a blue shift, leading to greater energy absorption by the nanoparticles (83). Given this increased energy absorption, a larger amount of radical formation from CAP can be anticipated when gold particles are present. Depending on the size and shape of these particles, the corresponding gold nanorods can be strongly irradiated in the presence of CAP.

The XRD analysis demonstrated that the lower intensity of the 2θ = 12 peak, assigned to the (001) plane, confirms the formation of GO through the Hummer method (84). The reduced intensity of this peak is attributed to the chemical interactions between the AuNPs and the GO platelets (85). Additionally, the peak at 2θ = 18⸰, corresponding to the (002) plane, is associated with the graphitic impurities in the GO samples (63). Two significant peaks at 24⸰ and 32⸰ indicate the successful doping of GO with AuNPs (64).

The optical absorbance analysis in the UV-Vis range revealed a redshift of approximately 7 nm from 230 nm, indicative of the reduction of GO by AuNPs (86). The absorption wavelengths between 200 - 219 nm confirmed close contact between gold and GO, facilitating efficient charge transfer between GO nanosheets and the metal (87). Furthermore, the redshift of the n → π* transitions in the C=O groups of GO from 300 nm to 314 nm confirmed the formation of reduced GO (71).

During the photocatalytic activity of AuNPs, electron bands are activated, and high-energy electrons are transferred to oxidizing agents, such as O₂, which are subsequently converted into radicals. These radicals can then attack organic molecules like biomolecules (88). In this process, the electrons are excited to higher energy levels (6sp bands), and upon interaction with O₂, radical species such as O_2_- are generated, which then combine with H^+^ to form other radicals like HO_2_• and OH•. In this study, the plasma provided the energy required to excite gold electrons to these higher energy states.

Other studies have highlighted the synergistic effect of non-thermal plasma and photocatalysis, which has shown enhanced CO removal from synthetic air (80% N_2_ + 20% O_2_) when using a combination of these two strategies (89). Additionally, UV radiation emitted by plasma plays a critical role in this process, complementing the effects of radical species. Helium/O₂ plasma generates radicals, electrons, and UV photons, all of which contribute to inducing apoptosis in cancer cells after being delivered to the tumor site.

Regarding the biological investigations, the non-doped GO nanoparticles did not significantly alter cell morphology, which could be attributed to their limited photoactivity. However, due to its π - π bonds, GO may exhibit some photocatalytic activity, generating free radicals (24). In contrast, the cell groups treated with the doped nanoparticles showed a more pronounced round cellular morphology compared to the regular polygonal shape, indicating the potential for apoptosis, which aligns with previous studies (73). The observations of cellular morphology also demonstrated that the distance of cells from the plasma tip had a more substantial effect on the cells than the exposure time. A similar study confirmed that cold plasma, with closer proximity and longer exposure, could induce greater cancer cell death due to the increased production of radical species under such conditions (90).

In all groups, except for those treated with the coupled nanoparticles, the cells were able to recover from the initial cell death over time, as their confluences increased after 72 hours. However, in the group with a 5 cm distance, no evidence of apoptosis was observed. This finding suggests that not only the type of nanoparticle affects cell fate, but the plasma conditions also play a crucial role. As a result, the groups treated with the composite nanoparticles lost their ability to induce cell death when exposed at longer distances from the plasma jet.

The MTT data further supported these findings, with all viability values of the non-doped group exceeding 50%, except for the group treated for 3 minutes at a distance of 3 cm. This clear difference highlights the stronger photoactivity of the doped nanoparticles compared to the non-doped GO particles. A related study reported an optimal distance of 1 cm and a treatment time of 5 minutes to achieve the IC_50_ point, further validating the enhanced efficacy of the doped nanoparticles under appropriate plasma conditions (91).

The observed differences in cell viability may be attributed to the presence of nanoparticles and the specific parameters of the cold plasma apparatus. Despite this, the difference in values reduced to less than 20%, 40%, and 16% after 24, 48, and 72 hours, respectively. These fluctuations over time support the notion of post-plasma cell death at 24 hours, followed by compensatory cell proliferation at 48 hours, and the induction of apoptotic gene expression at 72 hours. At longer plasma exposure durations, the photoactivity of the AuNPs was negligible, likely due to the overwhelming effect of the plasma. This finding contrasts with a related study, which reported about 34% cell death after 150 seconds of plasma irradiation (92). These discrepancies may be due to the differing quality of plasma devices used in various studies.

When comparing treatments at distances of 3 cm and 5 cm, it can be concluded that the effectiveness of plasma in inducing cell death diminishes with increased distance from the cells. This result is consistent with research showing that the bactericidal power of plasma weakens as the distance between the plasma nozzle and the target increases (93).

Regarding the toxicity of these nanoparticles, despite the high biocompatibility of graphene nanoparticles, they remain physiologically stable in various human cells (94) and bacteria (95). Other studies have shown that GO labeled with 188Re exhibited high stability in both pure water and cell culture media. In mice, approximately 67% of these nanosheets remained in the bloodstream 24 hours after injection, with no toxic effects observed when administered at a dose of 1 mg/kg of body weight for 14 days (96).

Another report emphasized the physiological stability of these nanoparticles within the body or in cells that had internalized them (97). The nanoparticles were dispersed in PBS, and their agglomerates were confirmed by dynamic light scattering (DLS). Similarly, in vivo conditions revealed that these particles formed protein coronas, which facilitated their distribution in media such as blood. Observations from atomic force microscopy (AFM) confirmed that the morphology of GO aggregates changed after the formation of coronas (98).

However, modifying these particles with hydrophilic compounds, such as manganese or dextran, may increase their stability in biological media or blood (99). Similar to the degradation of carbon nanotubes by neutrophils via a myeloperoxidase (MPO)-dependent mechanism, GO nanoparticles can also be degraded through this pathway. Despite their degradation, studies showed no toxicological effects or DNA damage in a bronchial epithelial cell line (100). Another study recommended using dispersants, suggesting that GO functionalized with polyethylene glycol (PEG) demonstrated the best biocompatibility at concentrations ranging from 3 mg/mL to 0.025 mg/mL on fibroblast cells (101).

AuNPs, known for their considerable stability against oxidation, have been proposed for clinical diagnostics and even therapeutic applications. After intravenous injection of AuNPs embedded in a gum-arabic matrix, the nanoparticles were detected within 30 minutes in various organs, including the heart, jejunum, brain, liver, spleen, kidney, and lung in juvenile swine. The lowest concentration was observed in the brain, while the liver showed the highest accumulation. After 24 hours, the concentration of the nanoparticles remained consistent with the levels observed after 30 minutes, indicating a strong affinity of AuNPs with tissue receptors (102).

Furthermore, smaller AuNPs demonstrated enhanced stability in animal models, such as mice. These nanoparticles were detectable in the blood and liver 72 hours post-injection, with the liver showing a significantly higher concentration compared to the blood (103). It has also been observed that smaller nanoparticles exhibit wider distribution across tissues, including the skin and intestines of rats (104). Similar to GO nanoparticles, the stabilization of AuNPs reduces their toxicity from mild to severe, as demonstrated in animal models (103).

### 5.1. Conclusions

In conclusion, for clinical trials involving GO-AuNPs, hydrophilic modifications are essential to minimize toxicity. Additionally, these modifications can facilitate the degradation of nanoparticles by reducing aggregation, thereby improving their safety and efficacy in medical applications.

The DPPH results were consistent with the findings from the cellular morphology and MTT assessments. The group exhibiting higher levels of apoptosis also showed increased radical production after plasma exposure. Previous studies have demonstrated that radical generation is an early event in the process of apoptosis, followed by the release of cytochrome c, caspase activation, and mitochondrial dysfunction (105). Based on our observations, longer plasma exposure appears to have a more pronounced effect than doping the GO nanoparticles with gold. Comparing the TCPS groups and the group treated with non-doped GO nanosheets, we can infer that GO nanoparticles may possess photothermal activity, likely due to their aromatic ring transition bonds, as noted in earlier studies (106).

Interestingly, radical production did not increase in the cells treated with nanoparticles in the absence of plasma. This result may be attributed to the higher biocompatibility of GO and AuNPs, particularly since both nanoparticles were evaluated earlier for their low toxicity, classified as first-grade toxicity. This outcome could also be due to the radical scavenging abilities of both doped and non-doped nanoparticles (107) in the absence of plasma exposure. Only in the group treated with the doped nanoparticles and plasma for 3 minutes at a distance of 3 cm was there a significant increase in local temperature compared to the TCPS group and the group treated with non-doped nanoparticles.

The precise mechanism by which cold plasma affects cells remains unclear, though several hypotheses are widely supported in the literature. Cold plasma generates radical species, including hydroxyl radicals, superoxide anions, alkoxyl radicals, and nitric oxides, along with non-radical species like hydrogen peroxide, ozone, and singlet O₂ (29). The interactions between these chemical compounds and cellular molecules are crucial, although the capacity of these species to induce apoptosis varies.

A proposed mechanism for the higher cell death observed after combination treatments of nanoparticles and cold plasma involves the absorption of ROS and RNS by the cells, leading to the selective killing of cancer cells. However, there is a critical concentration threshold for these nanoparticles, and the relationship between cell death and nanoparticle concentration is not linear (43). Another related mechanism involves a reduction in intracellular glutathione levels after treatment with both plasma and AuNPs, resulting in increased intracellular ROS levels and ultimately, cell death (30). Additionally, it has been demonstrated that the use of nanoparticles, such as gold, enhances cellular membrane permeability to radicals, triggering oxidative stress. This stress causes damage to proteins, lipids, and DNA (43), which initiates apoptosis through the activation of cellular death signals. Without AuNPs, the rate of cell death induced by radicals alone is insufficient.

Treatment with AuNPs makes cells more vulnerable to ROS and RNS, disrupting the balance between oxidant and antioxidant processes within the cells. Cancer cells, due to their higher metabolic rates, altered pH levels, and abnormal cellular activities, are particularly sensitive to DNA damage (108). Thus, the oxidative stress caused by radicals impacts cancer cells more profoundly than normal cells. It is well established that ROS can induce apoptosis, necrosis, senescence, or even cell cycle arrest (109). Among these radical species, ROS plays a major role in cell inactivation. Studies have shown that in the presence of ROS scavengers, the morphological and biological changes typically caused by plasma treatments are inhibited (110).

Furthermore, another mechanism of ROS-induced oxidative stress involves the upregulation of the p53 gene and p21 CKS inhibitor, which leads to cell cycle arrest and inhibits cell proliferation (111). A separate study showed that AuNPs coated with proteins can disrupt the clathrin-mediated endocytosis pathway, creating a brush-like layer on the cell surface that facilitates increased nanoparticle uptake. This enhanced uptake heightens the cell’s sensitivity to the radicals generated by cold plasma. In some cases, the effectiveness of both nanoparticles and cold plasma treatments is dependent on the specific cell type. For instance, normal cells such as astrocytes did not show significant nanoparticle diffusion across the cellular membrane, and their membrane morphology was minimally altered following cold plasma treatment (112).

The pattern of gene expression is a key factor in evaluating cellular responses to nanoparticles and cold plasma treatments. A related study using iron nanoparticles and CAP found a significantly lower expression of Bcl2, though no notable difference was observed when compared to other groups (22). In the present study, the expression of Bcl2 in the group treated with doped nanoparticles and plasma for 60 seconds was significantly lower, while Bax expression showed no significant difference in some groups under different conditions. The similar expression levels of Bax in both doped and non-doped groups treated with 30 seconds of plasma can be attributed to the photoactivity of these nanoparticles, largely due to π-π transitions associated with aromatic C−C single bonds (113).

Regarding the p53 gene, the current findings differ from a previous study that reported a time-dependent increase in p53 expression following CAP treatment (114). In contrast, p53 expression in the current study did not significantly change when plasma treatment time increased from 30 to 60 seconds. However, the p53 expression in both groups was significantly higher than in the remaining groups (P < 0.05), suggesting a specific role of phototherapy in regulating p53 expression. The positive effect of GO nanoparticles on gene expression was consistent, although the 60-second plasma treatment group showed similar expression levels to the 30-second group (P ≤ 0.05).

Additionally, the higher expression of caspase 3 compared to caspase 8 suggests that caspase 3 cleavage occurred as a result of caspase 8 activation following plasma treatment. This observation aligns with a study investigating CAP-mediated photothermal therapy in the presence of AuNPs (17). As in a previous report, caspase 8 expression remained stable following plasma treatment in the present study (115).

This study investigated the therapeutic potential of photocatalytic AuNPs in treating cervical cancer. The results demonstrated a noticeable increase in round, detached cells in the treated HeLa cells, especially when plasma treatment was applied at closer proximity and for longer durations. The nanoparticle concentrations exhibited toxicity at higher levels, with the biocompatible amounts being determined to optimize CAP conditions. Plasma treatment at a distance of 3 cm for 1 minute resulted in higher expression levels of apoptotic markers such as p53, Bax, and caspase 3/8, along with lower expression of Bcl2. These findings emphasize the importance of optimizing both plasma distance and exposure time. Future work should focus on developing precise tools to target plasma energy specifically to cancerous tissues with fine-tuned cold plasma parameters.

ijpr-23-1-150385-s001.pdf

## Data Availability

No new data were created or analyzed in this study. Data sharing is not applicable to this article.
