# Supplementary material for: Apoptotic Impact of Heliox Cold Plasma on a Cervical Cell Line Using Gold Nanoparticle-Doped Graphene Oxide Nanosheets
Source: Iran J Pharm Res. 2024 Nov 12;23(1):e150385. doi: 10.5812/ijpr-150385 (PMC11892752; doi:10.5812/ijpr-150385)
Supplement: ijpr-23-1-150385-s001.pdf [file ijpr-23-1-150385-s001.pdf]

For P53 expression:

Groups	TCPS-30 s	TCPS-60 s	GO-30 s	GO-60 s	GO/Au-30 s	GO/Au-60 s
TCPS-30 s		p-value≤0.05	p-value≤0.05	p-value≤0.05	p-value≤0.05	p-value≤0.05
TCPS-60 s			p-value≤0.05	p-value≤0.05	p-value≤0.05	p-value≤0.05
GO-30 s				p-value≤0.05	p-value≤0.05	p-value≤0.05
GO-60 s					p-value≤0.05	p-value≤0.05
GO/Au-30 s						p-value≤0.05

For BCL2 expression:

Groups	TCPS-30 s	TCPS-60 s	GO-30 s	GO-60 s	GO/Au-30 s	GO/Au-60 s
TCPS-30 s		p-value≤0.05	p-value≤0.05	p-value≤0.05	p-value≤0.05	p-value≤0.05
TCPS-60 s			p-value≤0.05	p-value>0.05	p-value≤0.05	p-value≤0.05
GO-30 s				p-value≤0.05	p-value≤0.05	p-value≤0.05
GO-60 s					p-value≤0.05	p-value≤0.05
GO/Au-30 s						p-value≤0.05

For Bax expression:

Groups	TCPS-30 s	TCPS-60 s	GO-30 s	GO-60 s	GO/Au-30 s	GO/Au-60 s
TCPS-30 s		p-value≤0.05	p-value≤0.05	p-value≤0.05	p-value≤0.05	p-value≤0.05
TCPS-60 s			p-value≤0.05	p-value≤0.05	p-value≤0.05	p-value≤0.05
GO-30 s				p-value≤0.05	p-value>0.05	p-value>0.05
GO-60 s					p-value≤0.05	p-value≤0.05
GO/Au-30 s						p-value>0.05

For Cas3 expression:

Groups	TCPS-30 s	TCPS-60 s	GO-30 s	GO-60 s	GO/Au-30 s	GO/Au-60 s
TCPS-30 s		p-value≤0.05	p-value≤0.05	p-value≤0.05	p-value≤0.05	p-value≤0.05
TCPS-60 s			p-value≤0.05	p-value≤0.05	p-value≤0.05	p-value≤0.05
GO-30 s				p-value≤0.05	p-value≤0.05	p-value≤0.05
GO-60 s					p-value≤0.05	p-value≤0.05
GO/Au-30 s						p-value≤0.05

For Cas8 expression:

Groups	TCPS-30 s	TCPS-60 s	GO-30 s	GO-60 s	GO/Au-30 s	GO/Au-60 s
TCPS-30 s		p-value≤0.05	p-value≤0.05	p-value≤0.05	p-value≤0.05	p-value≤0.05
TCPS-60 s			p-value≤0.05	p-value≤0.05	p-value≤0.05	p-value≤0.05
GO-30 s				p-value≤0.05	p-value≤0.05	p-value≤0.05
GO-60 s					p-value≤0.05	p-value≤0.05
GO/Au-30 s						p-value≤0.05
